# Supplementary material for: Genetic Diversity and Population Structure of Chinese Chestnut (Castanea mollissima Blume) Cultivars Revealed by GBS Resequencing
Source: Plants (Basel). 2022 Dec 14;11(24):3524. doi: 10.3390/plants11243524 (PMC9781913; doi:10.3390/plants11243524)
Supplement: Supplementary file 1 [file plants-11-03524-s001.zip › plants-1963162-supplementary.pdf]

Supplementary Materials: The following supporting information can be downloaded at: www.mdpi.com/xxx/s1, Table S1. Source information and ADMIXTURE results (k = 2) of 185 Chinese Chestnut landraces; Table S2. Outliers of the phylogenetic analysis; Figure S1. K values in the ADMIXTURE analysis of 185 Chinses chestnut landraces.

Supplementary Table S1. Source information and ADMIXTURE results (k=2) of 185 Chinese Chestnut landraces.

Table S1. Source information and ADMIXTURE results (k=2) of 185 Chinese Chestnut landraces.

Sample No.	Clustering based on ADMIXTURE	Grouping based on sources	North–South division based on sources	Origin
RISF-10	1	North of the Yellow River	North of the Yellow River	Changping, Beijing
RISF-11	1	North of the Yellow River	North of the Yellow River	Changping, Beijing
RISF-12	1	North of the Yellow River	North of the Yellow River	Huairou, Beijing
RISF-13	1	North of the Yellow River	North of the Yellow River	Huairou, Beijing
RISF-14	1	North of the Yellow River	North of the Yellow River	Huairou, Beijing
RISF-15	1	North of the Yellow River	North of the Yellow River	Changli, Hebei
RISF-17	1	North of the Yellow River	North of the Yellow River	Qian'an, Hebei
RISF-18	1	North of the Yellow River	North of the Yellow River	Qian'an, Hebei
RISF-19	1	North of the Yellow River	North of the Yellow River	Qian'an, Hebei
RISF-21	1	North of the Yellow River	North of the Yellow River	Zunhua, Hebei
RISF-22	1	North of the Yellow River	North of the Yellow River	Zunhua, Hebei
RISF-23	1	North of the Yellow River	North of the Yellow River	Zunhua, Hebei
RISF-128	1	North of the Yellow River	North of the Yellow River	Huairou, Beijing
RISF-129	1	North of the Yellow River	North of the Yellow River	Huairou, Beijing
RISF-130	1	North of the Yellow River	North of the Yellow River	Miyun, Beijing
RISF-133	1	North of the Yellow River	North of the Yellow River	Zunhua, Hebei
RISF-134	1	North of the Yellow River	North of the Yellow River	Zunhua, Hebei
RISF-135	1	North of the Yellow River	North of the Yellow River	Huairou, Beijing
RISF-136	1	North of the Yellow River	North of the Yellow River	Zunhua, Hebei
RISF-138	1	North of the Yellow River	North of the Yellow River	Zunhua, Hebei
RISF-139	1	North of the Yellow River	North of the Yellow River	Huairou, Beijing
RISF-140	1	North of the Yellow River	North of the Yellow River	Yanshan, Hebei
RISF-141	1	North of the Yellow River	North of the Yellow River	Huairou, Beijing
RISF-146	1	North of the Yellow River	North of the Yellow River	Huairou, Beijing
RISF-147	1	North of the Yellow River	North of the Yellow River	Changping, Beijing
RISF-148	1	North of the Yellow River	North of the Yellow River	Huairou, Beijing
RISF-150	1	North of the Yellow River	North of the Yellow River	Huairou, Beijing
RISF-151	1	North of the Yellow River	North of the Yellow River	Huairou, Beijing
RISF-1	1	Eastern Coastal region	North of the Yellow River	Guangde, Anhui
RISF-51	1	Eastern Coastal region	North of the Yellow River	Yixing, Jiangsu
RISF-52	1	Eastern Coastal region	North of the Yellow River	Haiyang,Shandong
RISF-56	1	Eastern Coastal region	North of the Yellow River	Junan, Shandong
RISF-57	1	Eastern Coastal region	North of the Yellow River	Junan, Shandong

RISF-58	1	Eastern Coastal region	North of the Yellow River	Junan, Shandong
RISF-59	1	Eastern Coastal region	North of the Yellow River	Junan, Shandong
RISF-61	1	Eastern Coastal region	North of the Yellow River	Laixi, Shandong
RISF-68	1	Eastern Coastal region	North of the Yellow River	Rushan, Shandong
RISF-71	1	Eastern Coastal region	North of the Yellow River	Tai'an, Shandong
RISF-73	1	Eastern Coastal region	North of the Yellow River	Tai'an, Shandong
RISF-74	1	Eastern Coastal region	North of the Yellow River	Tai'an, Shandong
RISF-78	1	Eastern Coastal region	North of the Yellow River	Tai'an, Shandong
RISF-79	1	Eastern Coastal region	North of the Yellow River	Tai'an, Shandong
RISF-80	1	Eastern Coastal region	North of the Yellow River	Tai'an, Shandong
RISF-81	1	Eastern Coastal region	North of the Yellow River	Tai'an, Shandong
RISF-82	1	Eastern Coastal region	North of the Yellow River	Tai'an, Shandong
RISF-84	1	Eastern Coastal region	North of the Yellow River	Tancheng, Shandong
RISF-85	1	Eastern Coastal region	North of the Yellow River	Tancheng, Shandong
RISF-87	1	Eastern Coastal region	North of the Yellow River	Yantai, Shandong
RISF-88	1	Eastern Coastal region	North of the Yellow River	Yantai, Shandong
RISF-89	1	Eastern Coastal region	North of the Yellow River	Zhaoyuan, Shandong
RISF-101	1	Eastern Coastal region	North of the Yellow River	Xinyi, Jiangsu
RISF-116	1	Eastern Coastal region	North of the Yellow River	Jiande, Zhejiang
RISF-131	1	Eastern Coastal region	North of the Yellow River	Tai'an, Shandong
RISF-132	1	Eastern Coastal region	North of the Yellow River	Tai'an, Shandong
RISF-137	1	Eastern Coastal region	North of the Yellow River	Rizhao, Shandong
RISF-31	1	Yangtze River Basin region	North of the Yellow River	Jingshan, Hubei
RISF-32	1	Yangtze River Basin region	North of the Yellow River	Jingshan, Hubei
RISF-36	1	Yangtze River Basin region	North of the Yellow River	Luotian, Hubei
RISF-111	1	Yangtze River Basin region	North of the Yellow River	Shucheng, Anhui
RISF-167	1	Yangtze River Basin region	North of the Yellow River	Luotian, Hubei
RISF-40	1	South Central region	North of the Yellow River	Shaoyang, Hunan
RISF-110	1	South Central region	North of the Yellow River	Heyuan, Guangdong
RISF-126	1	South Central region	North of the Yellow River	Liuyang, Hunan
RISF-145	1	South Central region	North of the Yellow River	Eshan, Yunnan
RISF-28	1	Midwest region	North of the Yellow River	Luoshan, Henan
RISF-92	1	Midwest region	North of the Yellow River	Chang'an, Shaanxi
RISF-170	1	Midwest region	North of the Yellow River	Chang'an, Shaanxi
RISF-8	2	North of the Yellow River	South of the Yellow River	Changping, Beijing
RISF-9	2	North of the Yellow River	South of the Yellow River	Changping, Beijing
RISF-16	2	North of the Yellow River	South of the Yellow River	Changli, Hebei
RISF-20	2	North of the Yellow River	South of the Yellow River	Qianxi, Hebei
RISF-24	2	North of the Yellow River	South of the Yellow River	Zunhua, Hebei
RISF-25	2	North of the Yellow River	South of the Yellow River	Zunhua, Hebei
RISF-63	2	North of the Yellow River	South of the Yellow River	Linqing, Shandong
RISF-107	2	North of the Yellow River	South of the Yellow River	Luan County, Hebei
RISF-112	2	North of the Yellow River	South of the Yellow River	Zunhua, Hebei
RISF-115	2	North of the Yellow River	South of the Yellow River	Changping, Beijing
RISF-149	2	North of the Yellow River	South of the Yellow River	Huairou, Beijing
RISF-2	2	Eastern Coastal region	South of the Yellow River	Guangde, Anhui
RISF-3	2	Eastern Coastal region	South of the Yellow River	Ningguo, Anhui
RISF-41	2	Eastern Coastal region	South of the Yellow River	Pizhou, Jiangsu

RISF-42	2	Eastern Coastal region	South of the Yellow River	Wu County, Jiangsu
RISF-43	2	Eastern Coastal region	South of the Yellow River	Xinyi, Jiangsu
RISF-44	2	Eastern Coastal region	South of the Yellow River	Xinyi, Jiangsu
RISF-45	2	Eastern Coastal region	South of the Yellow River	Yixing, Jiangsu
RISF-46	2	Eastern Coastal region	South of the Yellow River	Yixing, Jiangsu
RISF-47	2	Eastern Coastal region	South of the Yellow River	Yixing, Jiangsu
RISF-48	2	Eastern Coastal region	South of the Yellow River	Yixing, Jiangsu
RISF-49	2	Eastern Coastal region	South of the Yellow River	Yixing, Jiangsu
RISF-50	2	Eastern Coastal region	South of the Yellow River	Yixing, Jiangsu
RISF-53	2	Eastern Coastal region	South of the Yellow River	Haiyang,Shandong
RISF-54	2	Eastern Coastal region	South of the Yellow River	Haiyang,Shandong
RISF-55	2	Eastern Coastal region	South of the Yellow River	Junan, Shandong
RISF-60	2	Eastern Coastal region	South of the Yellow River	Junan, Shandong
RISF-62	2	Eastern Coastal region	South of the Yellow River	Laixi, Shandong
RISF-64	2	Eastern Coastal region	South of the Yellow River	Linyi, Shandong
RISF-65	2	Eastern Coastal region	South of the Yellow River	Linyi, Shandong
RISF-66	2	Eastern Coastal region	South of the Yellow River	Linyi, Shandong
RISF-67	2	Eastern Coastal region	South of the Yellow River	Linyi, Shandong
RISF-69	2	Eastern Coastal region	South of the Yellow River	Rushan, Shandong
RISF-70	2	Eastern Coastal region	South of the Yellow River	Tai'an, Shandong
RISF-72	2	Eastern Coastal region	South of the Yellow River	Tai'an, Shandong
RISF-75	2	Eastern Coastal region	South of the Yellow River	Tai'an, Shandong
RISF-76	2	Eastern Coastal region	South of the Yellow River	Tai'an, Shandong
RISF-77	2	Eastern Coastal region	South of the Yellow River	Tai'an, Shandong
RISF-83	2	Eastern Coastal region	South of the Yellow River	Tai'an, Shandong
RISF-86	2	Eastern Coastal region	South of the Yellow River	Tancheng, Shandong
RISF-94	2	Eastern Coastal region	South of the Yellow River	Shangyu, Zhejiang
RISF-95	2	Eastern Coastal region	South of the Yellow River	Shangyu, Zhejiang
RISF-96	2	Eastern Coastal region	South of the Yellow River	Shangyu, Zhejiang
RISF-98	2	Eastern Coastal region	South of the Yellow River	Laixi, Shandong
RISF-99	2	Eastern Coastal region	South of the Yellow River	Xintai, Shandong
RISF-100	2	Eastern Coastal region	South of the Yellow River	Changxing, Zhejiang
RISF-102	2	Eastern Coastal region	South of the Yellow River	Zhuji, Zhejiang
RISF-103	2	Eastern Coastal region	South of the Yellow River	Yixing, Jiangsu
RISF-104	2	Eastern Coastal region	South of the Yellow River	Minhang, Shanghai
RISF-105	2	Eastern Coastal region	South of the Yellow River	Zhaoyuan, Shandong
RISF-108	2	Eastern Coastal region	South of the Yellow River	Xuancheng, Anhui
RISF-113	2	Eastern Coastal region	South of the Yellow River	Tai'an, Shandong
RISF-122	2	Eastern Coastal region	South of the Yellow River	Yixing, Jiangsu
RISF-153	2	Eastern Coastal region	South of the Yellow River	Guangde, Anhui
RISF-156	2	Eastern Coastal region	South of the Yellow River	Pan'an, Zhejiang
RISF-157	2	Eastern Coastal region	South of the Yellow River	Pan'an, Zhejiang
RISF-158	2	Eastern Coastal region	South of the Yellow River	Ningguo, Anhui
RISF-159	2	Eastern Coastal region	South of the Yellow River	Guangde, Anhui
RISF-163	2	Eastern Coastal region	South of the Yellow River	Guangde, Anhui
RISF-164	2	Eastern Coastal region	South of the Yellow River	Qufu, Shandong
RISF-166	2	Eastern Coastal region	South of the Yellow River	Liyang, Jiangsu
RISF-171	2	Eastern Coastal region	South of the Yellow River	Wuyi, Zhejiang

RISF-172	2	Eastern Coastal region	South of the Yellow River	Wu County, Jiangsu
RISF-173	2	Eastern Coastal region	South of the Yellow River	Shaoxing, Zhejiang
RISF-174	2	Eastern Coastal region	South of the Yellow River	Wuyi, Zhejiang
RISF-175	2	Eastern Coastal region	South of the Yellow River	Wuyi, Zhejiang
RISF-176	2	Eastern Coastal region	South of the Yellow River	Yongjia, Zhejiang
RISF-177	2	Eastern Coastal region	South of the Yellow River	Yongjia, Zhejiang
RISF-178	2	Eastern Coastal region	South of the Yellow River	Tiantai, Zhejiang
RISF-179	2	Eastern Coastal region	South of the Yellow River	Jiangshan, Zhejiang
RISF-180	2	Eastern Coastal region	South of the Yellow River	Lanxi, Zhejiang
RISF-183	2	Eastern Coastal region	South of the Yellow River	Songyang, Zhejiang
RISF-184	2	Eastern Coastal region	South of the Yellow River	Pan'an, Zhejiang
RISF-185	2	Eastern Coastal region	South of the Yellow River	Pan'an, Zhejiang
RISF-4	2	Yangtze River Basin region	South of the Yellow River	Shucheng, Anhui
RISF-5	2	Yangtze River Basin region	South of the Yellow River	Shucheng, Anhui
RISF-6	2	Yangtze River Basin region	South of the Yellow River	Shucheng, Anhui
RISF-7	2	Yangtze River Basin region	South of the Yellow River	Shucheng, Anhui
RISF-33	2	Yangtze River Basin region	South of the Yellow River	Jingshan, Hubei
RISF-34	2	Yangtze River Basin region	South of the Yellow River	Luotian, Hubei
RISF-35	2	Yangtze River Basin region	South of the Yellow River	Luotian, Hubei
RISF-37	2	Yangtze River Basin region	South of the Yellow River	Luotian, Hubei
RISF-97	2	Yangtze River Basin region	South of the Yellow River	Luotian, Hubei
RISF-109	2	Yangtze River Basin region	South of the Yellow River	Huanggang, Hubei
RISF-154	2	Yangtze River Basin region	South of the Yellow River	Dongzhi, Anhui
RISF-155	2	Yangtze River Basin region	South of the Yellow River	Shucheng, Anhui
RISF-160	2	Yangtze River Basin region	South of the Yellow River	Jinzhai, Anhui
RISF-161	2	Yangtze River Basin region	South of the Yellow River	Jinzhai, Anhui
RISF-168	2	Yangtze River Basin region	South of the Yellow River	Luotian, Hubei
RISF-181	2	Yangtze River Basin region	South of the Yellow River	Luotian, Hubei
RISF-182	2	Yangtze River Basin region	South of the Yellow River	Luotian, Hubei
RISF-38	2	South Central region	South of the Yellow River	Shaoyang, Hunan
RISF-39	2	South Central region	South of the Yellow River	Rucheng, Hunan
RISF-106	2	South Central region	South of the Yellow River	Yangshuo, Guangxi
RISF-114	2	South Central region	South of the Yellow River	Xiangtan, Hunan
RISF-117	2	South Central region	South of the Yellow River	Xiangtan, Hunan
RISF-118	2	South Central region	South of the Yellow River	Changsha, Hunan
RISF-119	2	South Central region	South of the Yellow River	Changsha, Hunan
RISF-120	2	South Central region	South of the Yellow River	Xintian, Hunan
RISF-121	2	South Central region	South of the Yellow River	Liuzhou, Guangxi
RISF-123	2	South Central region	South of the Yellow River	Liuyang, Hunan
RISF-124	2	South Central region	South of the Yellow River	Liuyang, Hunan
RISF-125	2	South Central region	South of the Yellow River	Chengbu, Hunan
RISF-127	2	South Central region	South of the Yellow River	Shimen, Hunan
RISF-142	2	South Central region	South of the Yellow River	Yiliang, Yunnan
RISF-143	2	South Central region	South of the Yellow River	Yiliang, Yunnan
RISF-144	2	South Central region	Sorth of the Yellow River	Yiliang, Yunnan
RISF-152	2	South Central region	South of the Yellow River	Jingzhou, Hunan
RISF-162	2	South Central region	South of the Yellow River	Wugang, Hunan
RISF-165	2	South Central region	South of the Yellow River	Shaoyang, Hunan

RISF-169	2	South Central region	South of the Yellow River	Yangshuo, Guangxi
RISF-26	2	Midwest region	South of the Yellow River	Luoshan, Henan
RISF-27	2	Midwest region	South of the Yellow River	Luoshan, Henan
RISF-29	2	Midwest region	South of the Yellow River	Luoshan, Henan
RISF-30	2	Midwest region	South of the Yellow River	Luoshan, Henan
RISF-90	2	Midwest region	South of the Yellow River	Zhashui,Shaanxi
RISF-91	2	Midwest region	South of the Yellow River	Zhashui,Shaanxi
RISF-93	2	Midwest region	South of the Yellow River	Chang'an, Shaanxi

Supplementary Table S2. Outliers of phylogenetic tree analysis.

Table S2. Outliers of the phylogenetic analysis.

Sample No.	Origin
RISF-149	Beijing
RISF-72	Shandong
RISF-24	Hebei
RISF-79	Shandong
RISF-82	Shandong
RISF-58	Shandong
RISF-28	Henan
RISF-101	Jiangsu
RISF-52	Shandong
RISF-128	Beijing
RISF-61	Shandong
RISF-150	Beijing
RISF-92	Shaanxi
RISF-170	Shaanxi
RISF-84	Shandong
RISF-1	Anhui
RISF-57	Shandong

Supplement figure (S1):

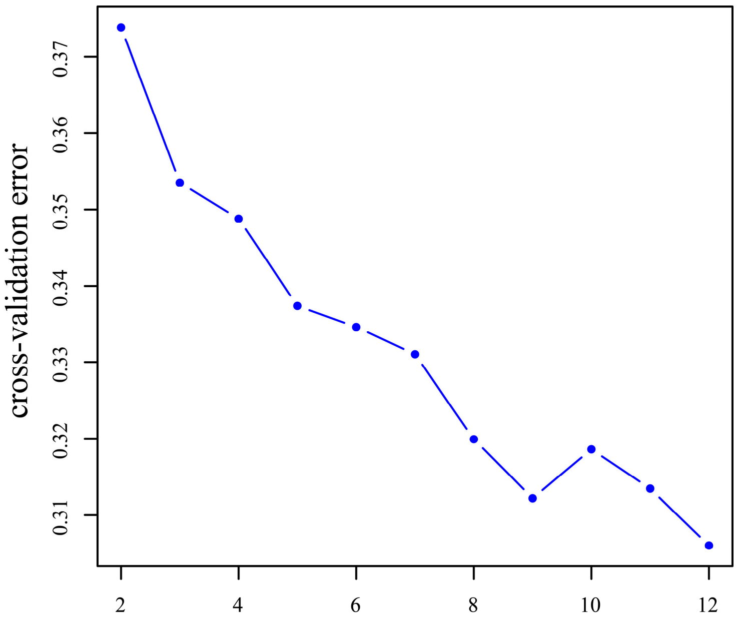

Figure S1. K values in the ADMIXTURE analysis of 185 Chinses chestnut landraces.
